# The sL1CAM in sera of patients with endometrial and ovarian cancers

**DOI:** 10.1007/s00404-016-4226-3

**Published:** 2016-11-10

**Authors:** Michał Wojciechowski, Ewa Głowacka, Miłosz Wilczyński, Anna Pękala-Wojciechowska, Andrzej Malinowski

**Affiliations:** 1 Department of Surgical, Endoscopic Gynecology and Oncology, Polish Mother’s Memorial Hospital-Research Institute, 281/283 Rzgowska St., 93-338 Łódź, Poland; 2 Department of Surgical and Endoscopic Gynecology, Medical University of Lodz, Łódź, Poland; 3 Department of Laboratory Diagnostics, Polish Mother’s Memorial Hospital-Research Institute, Łódź, Poland; 4 Clinical Pharmacology Department, Medical University of Lodz, Łódź, Poland

**Keywords:** sL1CAM, Ovarian carcinoma, Endometrial carcinoma

## Abstract

**Purpose:**

L1CAM is a cell adhesion molecule suspected to play an important role in carcinogenesis. The objective of the study was to evaluate the level of soluble L1CAM in the sera of patients with endometrial and ovarian carcinomas and verify the feasibility of the sL1CAM as a marker of these carcinomas.

**Methods:**

35 endometrial and 18 ovarian cancer patients were enrolled in the study. 43 patients with benign gynecological conditions constituted a control group. The sL1CAM serum level was measured with ELISA test in each patient and it was referred to the data from the surgical staging of the cancers.

**Results:**

The sL1CAM serum level was significantly lower in patients with endometrial cancer than in healthy women and slightly lower in the ovarian cancer group than in the control group. In the endometrial cancer group there was no correlation between sL1CAM concentration and cancer histopathology, stage or grade. sL1CAM concentration positively correlated with ovarian cancer stage and (not significantly) with grade.

**Conclusions:**

Despite the increasing data about the possible role of L1CAM as a strong prognostic factor of poor outcome in many cancers, we did not find evidence supporting the use of sL1CAM as a marker of endometrial or ovarian cancers.

## Introduction

Endometrial cancer is the most common cancer of the female genital tract in developed countries. Fortunately, it is diagnosed relatively quickly in many patients due to its early symptom—abnormal uterine bleeding. In the majority of cases, the cancer is discovered at FIGO stage I and presents endometrioid morphology (so-called “type 1” endometrial cancer) which can be cured in almost 90% of patients. About 10% of these patients, with potentially favorable prognosis, will however relapse and die from the disease. Some patients are diagnosed at more advanced stages or present with “type 2” papillary serous or clear cell endometrial cancer with substantially worse prognosis [1–3]. There is also a certain portion of uterine cancers of mixed morphology where the prognosis is particularly difficult to establish. The management of endometrial cancer consists of preoperative workup (dilatation and curettage, transvaginal ultrasonography, MRI, CT) followed by surgical staging (hysterectomy, bilateral salpingoophorectomy, pelvic lymphadenectomy in patients at high risk of relapse), which is meant to establish the final diagnosis, essential for prognosis and proper treatment [2–9]. These measures, however, are not sufficient to recognize the group of patients with early stage type 1 endometrial cancer, who, despite favorable prognosis and adequate treatment, will eventually die from the disease. In recent studies, L1CAM has been identified as a possible marker of poor prognosis and relapse in patients with endometrial cancer both of type 1, type 2 and mixed morphology [10–13].

Epithelial ovarian cancer remains one of the most frequent causes of cancer-related deaths in women. Due to lack of symptoms and no screening tests, it is seldom discovered at the early stages; therefore, the outcome is unfavorable in many cases. The management typically consists of primary debulking surgery, followed by platinum-based chemotherapy. Despite the treatment, often with complete response to therapy, the overall 5-year survival rate is disappointing and does not exceed 40% [14–16]. Therefore, there is a strong need for a marker which could either serve as a screening test and improve early detection or identification of the patients at high risk of chemoresistance and relapse. L1CAM again has been found to be a potential marker of poor outcome, short time to relapse and platinum resistance in ovarian cancer patients [10, 11, 17, 18].

L1CAM is a 200–220 kDa transmembrane adhesion molecule from the immunoglobulin family, consisting of an extracellular portion (six Ig-like domains with five fibronectin-type III repeats), a transmembrane part and a highly conservative cytoplasmatic tail [10, 19, 20]. Originally, it was discovered on neuronal cells and found to play a role in nervous system development [21]. Indeed, in healthy tissues, it is only expressed in collecting tubules in kidneys and peripheral nerve bundles. Hematopoietic cells such as B lymphocytes, T lymphocytes, and monocytes express rather low levels of L1CAM [11, 22]. It has also been found on the healthy ovarian surface epithelium [23]. It was however reported to be abnormally intensively expressed on many human cancer cells, including endometrial cancer, ovarian cancer, melanoma, colon adenocarcinoma positive to chromogranin, clear cell adenocarcinoma of the urinary bladder, pheochromocytoma, small cell lung carcinoma and tumors of the nervous system, and was identified as a possible marker of advanced stage, invasion and metastasis [22–26]. The regulation of its expression is not well understood and may be influenced by at least several mechanisms (demethylation of L1CAM promoter, TGF-β treatment, transcription factor SLUG overexpression or miR-34a expression) [12, 27, 28]. L1CAM can also be detected in its soluble form (sL1CAM) in the serum and ascites fluid from patients with ovarian, uterine and other cancers [10, 19, 24, 29–31]. The process of L1CAM cleavage, mediated by proteases, mainly ADAM10, enhances the ovarian and uterine cancer cell migration on various extracellular matrix components through autocrine/paracrine binding to integrins. This phenomenon may be responsible for accelerated tumor dissemination in L1CAM-positive tumors [10].

The hallmarks of carcinogenesis are progression of the primary tumor and formation of distant metastases, which demand substantial rearrangement in cell and tissue morphology [32]. Cell-to-cell and cell-to-matrix adhesion mediated by adhesive molecules of several families provide not only the structural support of the tissue, but also play an important role in the regulation of many processes such as proliferation, migration, angiogenesis, vascular sprouting and differentiation, which are essential for invasion and metastasis formation [32–38]. Some of the adhesive molecules, like E-cadherin, are responsible for homophilic intercellular interactions and proper tissue structure [39]. Others, like L1CAM, bind the cell to the matrix components during cell migration [12]. Formation of metastasis is believed to begin with loss of E-cadherin-dependent connections which allows the escape of the cell from its surrounding. It subsequently would migrate along the extracellular matrix components, which is mediated by other adhesive molecules such as CD44 or L1CAM [12, 19, 37, 38, 40, 41].

## L1CAM in endometrial and ovarian cancers

L1CAM is not expressed in normal endometrium [12, 22]. It had been believed to be absent on the ovarian surface epithelium, stromal cells of the ovary and oocytes until Zecchini et al. found it to be abundant on the ovarian surface epithelium [23]. It however may be highly expressed on the endometrial and ovarian cancer cell surface [10–12, 17, 23]. It is absent in the majority of the early-stage endometrioid endometrial cancer (type 1) cells and is usually strongly expressed on papillary serous and clear cell cancer (type 2) endometrial cells. Its expression negatively correlates with the expression of E-cadherin and estrogen/progesterone receptors, known markers of good prognosis. Thus, L1CAM-negative endometrial cancers tend to be E-cadherin and ER/PR positive [12]. However, there is a certain number of type 1 endometrioid endometrial cancers, positive for L1CAM as well as E-cadherin, but ER/PR negative and thus of type 2-like profile. L1CAM-positive cells may be found in the clear cell/papillary serous foci of the mixed endometrioid/non-endometrioid morphology cancers, which might facilitate identification of such small areas of differentiation within the endometrioid, L1CAM-negative background [12]. In the L1CAM-positive endometrial cancers, like in colon and ovarian cancers, the L1CAM may often be found at the leading edge of the cancer—the area, which also tends to be E-cadherin and ER/PR negative. It has been concluded that such similar and repeatable inverse correlation of L1CAM and E-cadherin and ER/PR receptors’ expression may suggest their participation in the process of epithelial–mesenchymal transition (EMT) [12, 23, 42, 43]. The EMT is a key process during tissue development as well as cancer progression, leading to the acquisition of fibroblast-like morphology of the epithelial cells, reduced intercellular interactions and enhanced motility [42]. As L1CAM is both abundantly present on the normal epithelium of the ovary and the surface of the cells of advanced ovarian cancer, it is suggested to play two opposite roles: in healthy epithelium it would support cell–cell adhesion and apoptosis, whereas in the transformed tissue it would inhibit apoptosis and intercellular interactions, and promote cell proliferation, invasion and transendothelial migration [23].

L1CAM expression is a marker of poor prognosis, short recurrence-free survival and advanced stage of the disease in many cancers including endometrial, ovarian carcinomas, pancreatic ductal adenocarcinoma melanoma and glioblastoma [23, 42]. Although it is not surprising in the case of “type 2” uterine cancers, strikingly, despite potentially good prognosis, type 1 endometrioid cancers, positive for L1CAM, behave in the same manner as type 2 cancers with poor prognosis and short time to recurrence [10, 12, 25, 26]. Similarly, in the ovarian cancer, L1CAM tends to be expressed at the advancing edge of the tumor and in all examined patients its expression correlates with high-grade histopathology (G3), advanced FIGO stage, risk of incomplete debulking at primary surgery, lymph node involvement as well as overall and disease-free survival [10, 23, 44]. It is significantly more expressed by cancers with impaired p53 function, which are believed to be more aggressive and resistant to apoptosis and chemotherapy [18, 44]. What is important, the poor clinical outcome for patients with L1CAM-positive ovarian cancers is similar irrespective of the tumor histological type [10]. Due to its expression being specific to the Mullerian tract-derived cancers like ovarian and endometrial cancer, L1CAM has been suggested as a possible marker differentiating those carcinomas from cases of metastatic cancer of unknown primary site in women [45].

As already mentioned, L1CAM expressed on the surface of cancer cells is released to the body fluids and may be found in serum or ascites fluid of endometrial or ovarian cancer patients, as well as in the culture medium of many human and mouse L1-positive carcinoma cell lines [10, 19, 24, 46]. In ovarian cancer, the L1CAM cleavage intensity is a function of L1CAM surface expression and has been found to be a marker of poor progression-free survival and chemoresistance, although by itself it probably cannot rescue the cells from apoptosis [17, 18, 44]. Several mechanisms are responsible for this phenomenon. One of them is a direct, membrane-proximal cleavage of the extracellular part mainly by ADAM10 protease, which creates ~200 kDa sL1CAM soluble form [19, 24, 46]. The other mechanism, probably predominant in the ovarian cancer, is secretion of sL1CAM in secretory vesicles—exosomes and apoptotic membrane vesicles [24].

It is still unclear whether and to what extent the biological effects of L1CAM are mediated by the soluble form or full-length, membrane-bound particle [42]. The full-length L1CAM exerts its biological role via several signaling pathways, depending on the substrate attached [42]. The sL1CAM is bound by neurocan—a proteoglycan of the extracellular matrix, which stimulates integrin-mediated cell migration [30, 46] or directly stimulates the cell migration on fibronectin and laminin by autocrine binding to aνβ5 integrin [19, 24, 42]. Thus, sL1CAM promotes cancer progression. It was also found to protect cancer cells from apoptosis in vitro [18, 19]. sL1CAM binds to the integrins on endothelial cells exerting a proangiogenic effect, which is crucial for cancer invasion and may be inhibited by anti-sL1CAM antibodies [29]. The process of sL1CAM shedding has been shown to be involved in the acquisition of chemoresistance by ovarian cancer cells [18] and correlates with progression-free survival (independent prognostic marker) and overall survival of the ovarian cancer patients [44]. There are suggestions that sL1CAM could serve better than Ca125 in the surveillance of free-of-disease ovarian cancer patients and in searching for recurrence [11]. Such a possible role for sL1CAM as a marker of poor prognosis has been proposed for gastrointestinal stromal tumors [31].

This would suggest that L1CAM expression is invariably implicated in cancer progression-related processes that highly negatively influence the course of the disease. This makes it a potent marker of clinical outcome in ovarian and endometrial cancers, which potentially could modify the diagnostic and therapeutic approach. In a recent multicenter study, L1CAM has been called “the best ever published prognostic factor in FIGO stage I, type I endometrial cancers” [13].

The aim of this study is to assess the concentration of soluble forms of L1CAM in sera of patients with endometrial and ovarian cancer and verify the feasibility of sL1CAM as a marker of the disease and its correlation with clinicopathological parameters of the disease.

## Materials and methods

35 patients with endometrial cancer and 18 with ovarian cancer were operated on in 2013 in the Department of Endoscopic and Surgical Gynecology and Oncological Gynecology, Polish Mother’s Memorial Hospital-Research Institute, Łódź, Poland. After the informed consent was obtained, the peripheral blood samples were collected, allowed to clot and centrifuged. Serum was stored at −20 °C. The concentration of soluble forms of L1CAM was assessed with ELISA Uscn E90959Hu set. The clinical data including the histological type of the cancer, the grading and the staging according to the FIGO 2009 were collected after the surgery. These data were matched with the L1CAM serum concentration. Similarly, after the informed consent was obtained, the sera of 43 volunteer patients with benign gynecological conditions were collected for L1CAM soluble form detection. This group served as the control group.

The statistical analysis was made with STATISTICA PL 10 and SPSS 21 software. The distribution of variables was checked with the Shapiro–Wilk test. The qualitative data correlation was verified with Chi-square and Chi-square test with Yates correction. The quantitative data were analyzed with ANOVA Kruskal–Wallis (for three groups) and non-parametrical *U* Mann–Whitney tests (when two groups were compared). Spearman rank correlation test was used to verify the association between two variables. *p* < 0.05 was considered to be significant.

## Results

35 patients with endometrial cancer and 18 patients with cancer of the ovary were included in the study as well as the group of 43 patients with benign gynecological conditions constituting the “control group”. The “ovarian cancer” group was significantly younger than the “endometrial cancer” group (54.9 vs. 63.8 years, *p* = 0.0493) and also younger than the control group (54.9 vs. 62.6 years, *p* = 0.0682).

The majority of patients with endometrial cancer (*n* = 22, 64.7%) presented with early stage disease (FIGO I, Table 1) of endometrioid morphology (*n* = 31, 88.6%, Table 2). Most of the patients with ovarian cancer (*n* = 13, 72.2%, Table 1) were in advanced stages (at least FIGO III), mainly of papillary serous morphology (*n* = 9, 50.0%, Table 2).

**Table 1:** FIGO staging of the cancers

Stage	Endometrial cancer		Ovarian cancer	
	*N*	%	*N*	%
1	22	64.7	5	27.8
2	1	2.9	0	0
3	8	23.5	13	72.2
4	3	8.8	0	0

**Table 2:** Tumor histopathology

Histopathology	Endometrial cancer		Ovarian cancer	
	*N*	%	*N*	%
Endometrioid adenocarcinoma (AE)	31	88.6	4	22.2
Papillary serous (PS)	2	5.7	9	50.0
AE + PS	1	2.9	1	5.6
Clear cell (CC)	0	0	3	16.7
AE + CC	1	2.9	0	0
Anaplastic carcinoma	0	0	1	5.6

The endometrial cancer was well differentiated in 16 (48.5%) patients (Table 3) whereas the ovarian cancer was poorly differentiated in 9 (50.0%) patients.

**Table 3:** Tumor grading

Grade	Endometrial cancer		Ovarian cancer	
	*N*	%	*N*	%
G1	16	48.5	3	16.7
G2	12	36.4	6	33.3
G3	5	15.2	9	50.0

There was an equal distribution of endometrial cancer patients with shallow (<1/2 of the depth) and deep (>1/2 of the depth) myometrial invasion (18 vs. 17 patients respectively, ns).

The serum sL1CAM concentration varied significantly between the groups (*p* = 0.0062, Table 4). It was significantly smaller in patients with endometrial cancer than in healthy women (*p* = 0.0043) and insignificantly smaller in the ovarian cancer group than in the control group (*p* = 0.603).

**Table 4:** The serum level of L1CAM

L1CAM	*N*	Average	Median	Min	Max	Q25	Q75	SD
Endometrial cancer	35	254.7	93.3	2.1	2652	55	169	505.42
Ovarian cancer	18	474.2	117.6	2.1	3800	58	289.6	953.33
Control group	43	321.4	175	53	1900	117	227	393.18

**Fig. 1:**
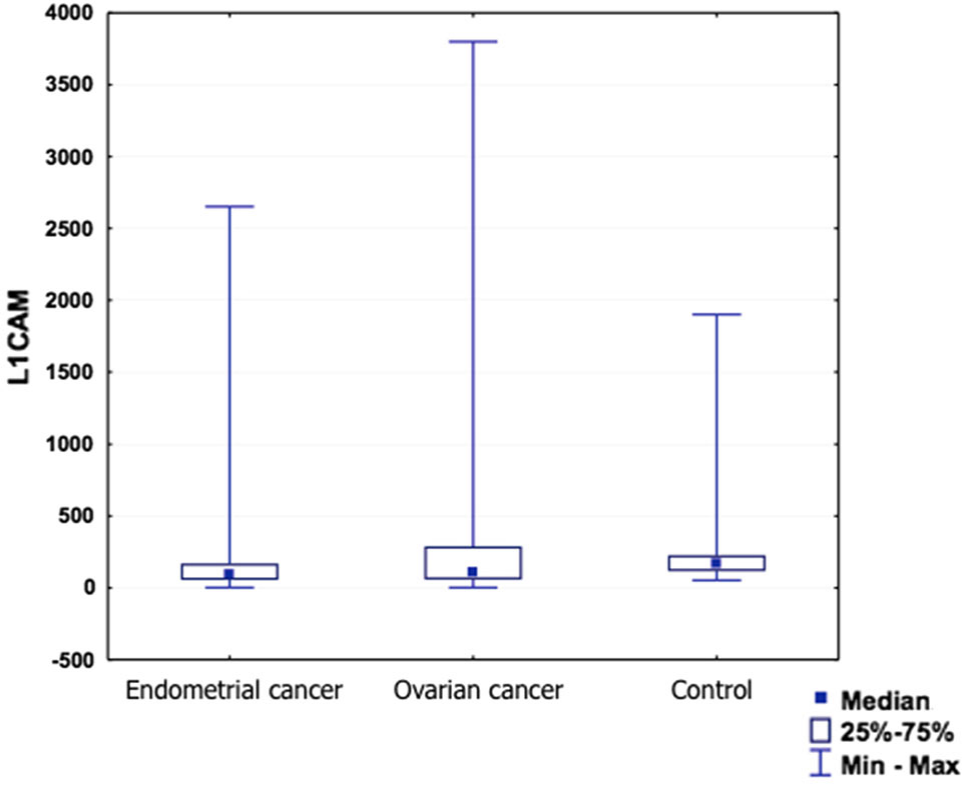
The serum level of sL1CAM

The results within the groups were, however, importantly spread out which made the analysis very difficult (Fig. 1).

**Fig. 2:**
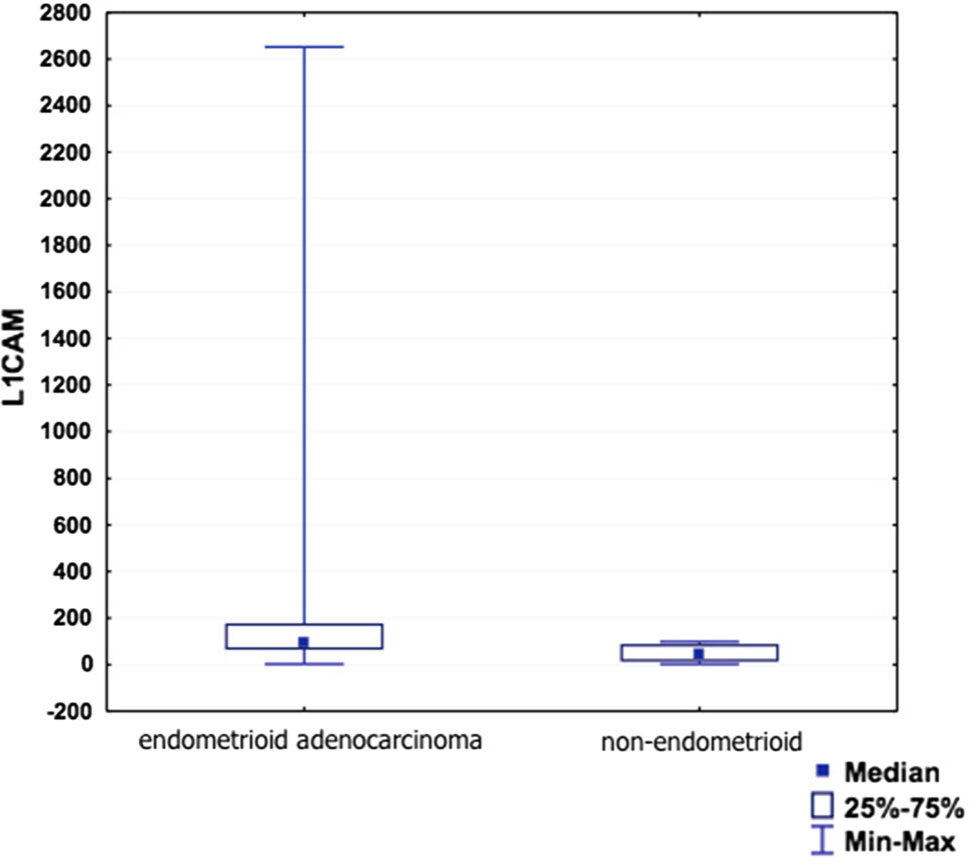
sL1CAM serum level in type 1 and type 2 endometrial carcinoma patients

Unfortunately, due to the small groups of patients, it was impossible to verify the correlation between the L1CAM concentration and endometrial or ovarian cancer histopathology. We divided the endometrial cancer patients into “type 1” (endometrioid adenocarcinoma) and “type 2” (non-endometrioid carcinoma) groups and compared them according to the sL1CAM concentration, but the difference turned out to be insignificant. Therefore, the sL1CAM serum concentration did not prove useful in terms of identification of patients with more aggressive, “type 2” cancer morphology (Fig. 2).

In the endometrial cancer group, we have not found any correlation between sL1CAM concentration and tumor stage or grade, or depth of myometrial invasion.

**Fig. 3:**
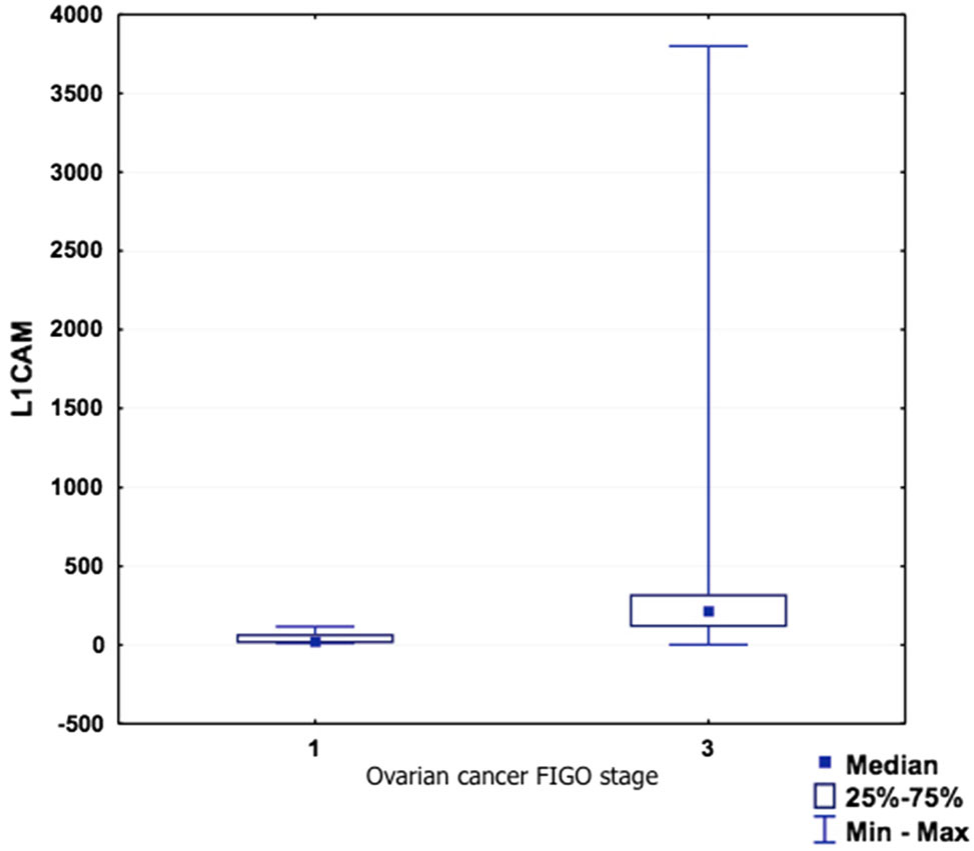
sL1CAM and ovarian cancer stage

**Fig. 4:**
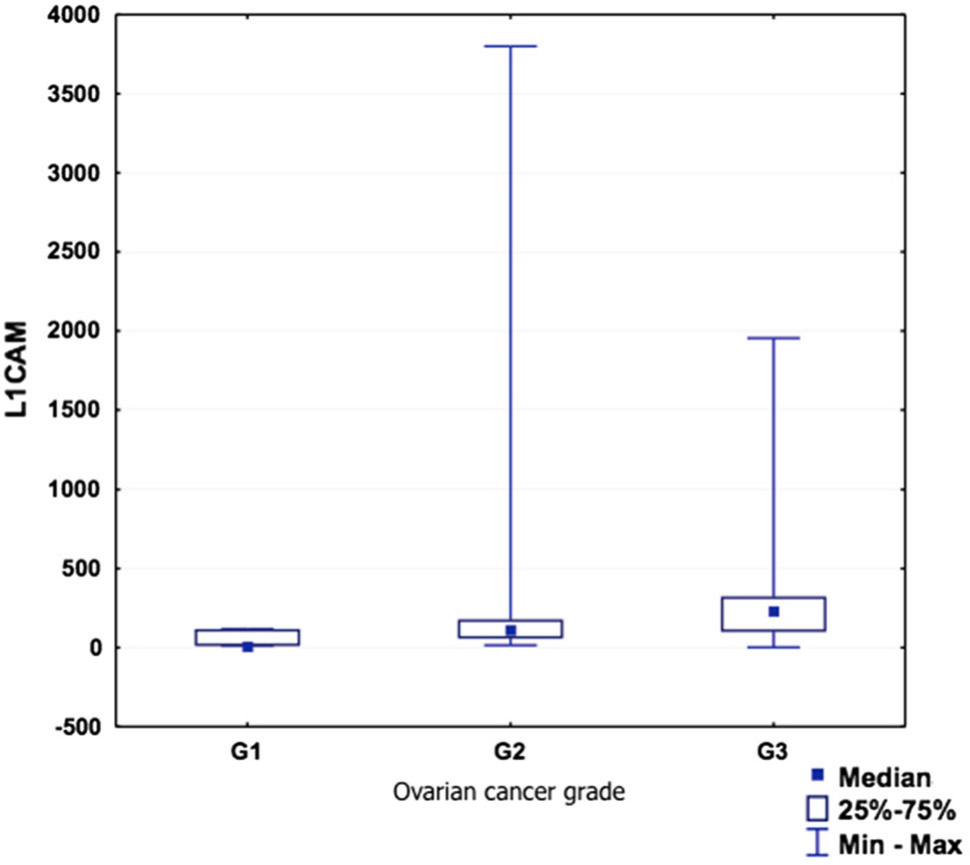
sL1CAM and ovarian cancer grade

L1CAM concentration positively correlated with ovarian cancer stage (*p* = 0.0152, *R* = 0.5618). There is also a positive, but statistically insignificant correlation with ovarian cancer grade (*p* = 0.0968, *R* = 0.4159) (Table 5; Figs. 3, 4). Due to a small amount of patients with positive lymph nodes, we were unable to analyze the L1CAM expression in relation to the lymph node status.

**Table 5:** sL1CAM and endometrial and ovarian cancer stage and grade

	*N*	Spearman	*p*
L1CAM and endometrial cancer stage	35	−0.1540	0.3844
L1CAM and endometrial cancer grade	35	−0.1137	0.5285
L1CAM and ovarian cancer stage	18	0.5618	0.0152
L1CAM and ovarian cancer grade	18	0.4159	0.0968

We compared the sL1CAM concentration according to distant metastases formation. It turned out to be more expressed in the control group than in cancer patients without metastases (taking both cancers together) (*p* < 0.01). Patients with distant metastases had significantly higher levels of sL1CAM than those without metastases (*p* < 0.05). Analyzing the cancers separately, we found significantly higher levels of sL1CAM in the control group than in endometrial cancer patients without metastases (*p* = 0.0352) and ovarian cancer patients without distant metastases (*p* = 0.0242). Ovarian cancer patients with disseminated disease had higher sL1CAM levels than those without metastases (*p* = 0.0140) (Table 6).

**Table 6:** sL1CAM concentration and distant metastases (EC—endometrial cancer, OC—ovarian cancer)

Cancer with metastases > cancer without metastases	Cancer with metastases vs. control	Cancer without metastases < control	EC with metastases vs. EC without metastases	EC with metastases vs. control	EC without metastases < control	OC with metastases > OC without metastases	OC with metastases vs. control	OC without metastases < control
*p* = 0.044	ns.	*p* = 0.0056	ns.	ns.	*p* = 0.0352	*p* = 0.0140	ns.	*p* = 0.0242

## Discussion

Our investigation is a pilot study, which was supposed to give us an impression on how useful the detection of sL1CAM in serum of patients with uterine or ovarian carcinomas could be. Because it is a prospective study we could not assess the feasibility of sL1CAM as a prognostic factor in terms of survival yet. A vast majority of our endometrial cancer patients had pure endometrioid endometrial cancer (type I) (31 patients out of 35, 88.6%). Therefore, the type II cancer group was too small (four patients: two non-endometrioid and two mixed morphology) to be evaluated statistically. The sL1CAM concentration was weak and it was even significantly weaker in the sera of uterine cancer patients than in healthy controls group. It is surprising in view of most previously cited data, but as it was already mentioned only 17% of stage I endometrioid endometrial cancers and up to 28% of all uterine cancers including type II and advanced stages tumors express L1CAM in their cells [10, 13]. When we compared the sera of patients with FIGO stage I disease (23 patients) and more advanced stages (12 patients), the sL1CAM level turned out to be insignificantly higher in the letter group (367.1 vs. 200.2, *p* = 0.51), which could reflect a possible L1CAM positivity in this group. To our knowledge, the percentage of sL1CAM-positive sera among endometrial cancer patients has not been investigated yet. We did not do an immunohistochemical evaluation of cancer specimens for L1CAM membranous expression; thus, we cannot say about the proportion of L1CAM-positive cancers in our group. The same applies to the ovarian cancer group. It has been shown in previous reports that L1CAM is expressed on every ovarian surface epithelium cell, whereas it appears only on a subset of ovarian cancer cells, mainly of advanced stages [23]. To reach conclusive results, a greater number of endometrial cancer patients needs to be investigated for sL1CAM serum level. We therefore could not assess the correlation between the L1CAM serum level and endometrial cancer histopathological type, due to a relatively small group of non-endometrioid cancers. There was no correlation of the L1CAM serum level and tumor stage or grade. Again, this could seem surprising in view of recent data suggesting L1CAM to be the most reliable prognostic factor in endometrial cancer. It has to be remembered, however, that these data apply to the membranous expression of the adhesive molecule and have not been analyzed yet in relation to the expression of its soluble forms.

In the ovarian cancer group, the sL1CAM serum level was also slightly lower than in the healthy controls (although not statistically significant, *p* = 0.608). Although surprising, this result is in line with the report of Zecchini et al. [23]. Moreover, as the sL1CAM may be present in the ascites fluid from ovarian cancer patients via multiple mechanisms, such as direct cleavage by proteases or in secretory vesicles [24], it is possible that in the sera of these patients there also exists a certain heterogeneity of sL1CAM forms. This heterogeneity could affect the sL1CAM detection by the antibodies we used. Among the patients with ovarian cancer, the serum sL1CAM concentration significantly positively correlated (*p* = 0.015) with ovarian cancer FIGO stage, and slightly positively (although without significance, *p* = 0.096) with tumor grade (G). These results might reflect an increasing L1CAM expression on the surface of ovarian cancer cells in patients with more advanced and aggressive disease [23]. Again, like in the previous group, we have not analyzed the cell membrane expression of L1CAM in ovarian cancer patients and we cannot express the percentage of L1CAM-positive cancers among our patients. As it was mentioned, in ovarian cancer, the L1CAM shedding is a function of its surface expression [44].

Only one patient with endometrial cancer had positive lymph nodes, so that statistic evaluation was impossible. sL1CAM turned out to be more expressed in patients with distant metastases (taking all cancers into account) than without (*p* < 0.05) and in the control group than in the cancer group without metastases (*p* < 0.01). There was no difference between the control group and the cancer group with metastases. The same pattern was observed when the cancers were analyzed separately: both endometrial and ovarian cancer patients without metastases had lower levels of sL1CAM than healthy patients. The level of sL1CAM seemed to rise again when distant metastases appeared, as it was the case in the ovarian cancer group. There were too few patients with endometrial cancer and distant metastases for such analysis. This again might reflect the L1CAM participation in tumor progression and its re-expression and cleavage in the more advanced stages. The most probable explanation is that, according to previous reports, L1CAM cleavage is a function of its surface expression. As it is abundantly expressed on healthy ovarian epithelium and reappears only on advanced ovarian cancer cells, the serum sL1CAM concentration reflects this pattern. The molecule becomes again detectable in patients with advanced disease. If it was the case, sL1CAM could not be an early marker of the disease, but rather a marker of an advanced stage of the cancers.

To conclude, despite the increasing data about the possible role of L1CAM as a strong prognostic factor of poor outcome in many cancers, including endometrial and ovarian cancer as well as the promising data concerning the possibility of detection of L1CAM soluble forms in sera of cancer patients, we did not find evidence for sL1CAM feasibility as a marker of endometrial or ovarian cancers.

We believe a study including more numerous groups of patients could reveal more conclusive results and verify our findings.
